# Acral malignant melanoma; emphasis on the primary metastasis and the usefulness of preoperative ultrasound for sentinel lymph node metastasis

**DOI:** 10.1038/s41598-019-52180-y

**Published:** 2019-11-04

**Authors:** Mi-ri Kwon, Sang-Hee Choi, Kee-Taek Jang, Jung-Han Kim, Goo-Hyun Mun, Jeeyun Lee, Dong-Youn Lee

**Affiliations:** 1 Department of Radiology, Samsung medical center, Sungkyunkwan University School of Medicine, Seoul, South Korea; 2 Department of Pathology, Samsung medical center, Sungkyunkwan University School of Medicine, Seoul, South Korea; 3 Division of Endocrine Surgery, Department of Surgery, Samsung medical center, Sungkyunkwan University School of Medicine, Seoul, South Korea; 4 Department of Plastic surgery, Samsung medical center, Sungkyunkwan University School of Medicine, Seoul, South Korea; 5 Division of Hematology/Oncology, Department of Medicine, Samsung medical center, Sungkyunkwan University School of Medicine, Seoul, South Korea; 6 Department of Dermatology, Samsung medical center, Sungkyunkwan University School of Medicine, Seoul, South Korea

**Keywords:** Health care, Medical research

## Abstract

This study aimed to evaluate the most common primary metastatic location of acral malignant melanoma and to evaluate the usefulness of preoperative ultrasound for sentinel lymph node metastasis. Ninety-eight Korean acral malignant melanoma patients were enrolled. Acral malignant melanoma was present in 76 lower limbs and in 22 upper limbs. The most common origin location was the sole (33.7%). The most common location of primary metastasis was loco-regional recurrence (22/34, 64.7%). The sensitivity, specificity, positive predictive value, and negative predictive value of preoperative sentinel lymph node ultrasound was 29.1%, 94.6%, 63.6%, and 80.5%, respectively. We postulate the unusefulness of preoperative ultrasound for sentinel lymph node metastasis in acral malignant melanoma.

## Introduction

In Asian populations, cutaneous melanomas are relatively rare and manifest differently from Caucasian populations, commonly occurring in acral locations including hands and feet^1,2^. Delayed diagnosis due to the location and the unfamilialities of the disease made the thickness of tumors become thicker and the diagnosis be done in advanced stage^3^.

Many variables have been investigated for evaluating prognostic value of acral malignant melanoma. Some studies reported that tumor thickness, the presence of ulceration, and mitotic rate were powerful predictor of survival of acral malignant melanoma, not different from melanoma in western countries^4–8^. The involvement of sentinel lymph node (SLN) is one of the most significant prognostic factors in recurrence and survival in melanoma patients^6,7,9,10^.

As a whole, primary cutaneous melanomas metastasize most frequently to the regional lymph nodes. Also, tumors located in the extremities had the strongest predilection to develop in-transit metastases^11^.

To evaluate SLN metastasis, SLN biopsy (SLNB) using lymphoscintigraphy with hand-held gamma probe in operating room is widely used^9,12^. The preoperative ultrasound (US), Doppler study, US-guided fine needle aspiration or core biopsy has been introduced to support the SLNB^13–18^. We tried to investigate the usefulness of preoperative US to observe SLN metastasis in acral malignant melanoma. Previous studies evaluating the value of preoperative US have been inconsistent, because they are included all origins of cutaneous melanoma. We are going to enroll only acral malignant melanoma^13–15^.

Therefore, the purposes of this study were retrospectively to document the most common primary metastatic site of acral malignant melanoma and to investigate the usefulness of preoperative US for SLN metastasis prior to operative resection of SLN in acral malignant melanoma.

## Results

Of the 150 patients diagnosed with acral melanoma, 52 were excluded because of no available pathologic information about tumor thickness (n = 1), no available pathologic information about LN metastasis (n = 41), synchronous melanoma in other sites or distant metastasis at initial work-up (n = 5), and follow-up loss (n = 5).

Table 1 shows detailed demographic and pathologic findings of patients. Seventy-six patients had lower limb acral malignant melanomas including the sole (n = 33), heel (n = 22), toe (n = 18, with 17 in the greater toe and 1 in the 5^th^ toe), nailbed (n = 2), and foot dorsum (n = 1). Twenty-two patients had upper limb acral melanomas including fingers (n = 16), palms (n = 3), and nailbeds (n = 3). The most common subtype was acral lentiginous growth in 91 patients (89.2%) followed by nodular growth in 7 patients (10.8%). Twenty-four patients of the 98 patients (24.5%) showed SLN involvement after SLN resection.

**Table 1:** Demographics and pathologic findings of melanoma in acral locations.

Characteristics	*n*	%
Sex		
male	51	52.0
female	47	48.0
Age (range) (years)	60 (27–88)	
Location		
palm	3	3.1
finger	16	16.3
finger nailbed	3	3.1
foot dorsum	1	1.0
heel	22	22.4
sole	33	33.7
toe	18	18.4
toe nailbed	2	2.0
Tumor thickness		
Tis	12	12.2
T1	15	15.3
T2	18	18.4
T3	27	27.6
T4	26	26.5
Ulceration	25	25.5
Perineural invasion	2	2.0
Endolymphatic invasion	3	3.1
Subtype		
acral lentiginous growth	91	92.9
nodular growth	7	7.1
Preoperative US		
negative	87	88.8
positive	11	11.2
LN result		
negative	74	75.5
positive	24	24.5

Thirty-four patients showed metastasis (34/98, 34.7%) during follow-up (median 21.4 months, range 1 to 69 months). The most common metastatic type was loco-regional recurrence (n = 22; 10 in regional LNs, 7 in in-transit metastases, 4 in both regional LN and in-transit metastases, and 1 local recurrence), followed by distant metastases (n = 12; 5 in lung, 4 in distant LNs, 1 in contralateral limb, 1 in scalp, and 1 in liver).

The sensitivity of preoperative US of SLNs was 29.2% (95% confidence intervals (CIs), 10.9% to 47.3%) and the specificity was 94.6% (89.4% to 99.8%). Positive predictive value (PPV), negative predictive value (NPV), and diagnostic accuracy were 63.6% (35.2% to 92.1%), 80.5% (72.1% to 88.8%), and 78.5% (70.4% to 86.7%), respectively (Table 2). The discrepancy between the preoperative US finding and SLN metastasis increased proportionally to tumor thickness (T staging) with statistically significance (*p* < 0.001).

**Table 2:** Cross table showing the relationship between the US findings and SLN metastasis.

		Preoperative US		Total
		positive	negative	
SLN metastasis	Positive	7	17	24
	negative	4	70	74
Total		11	87	98

**Figure 1:**
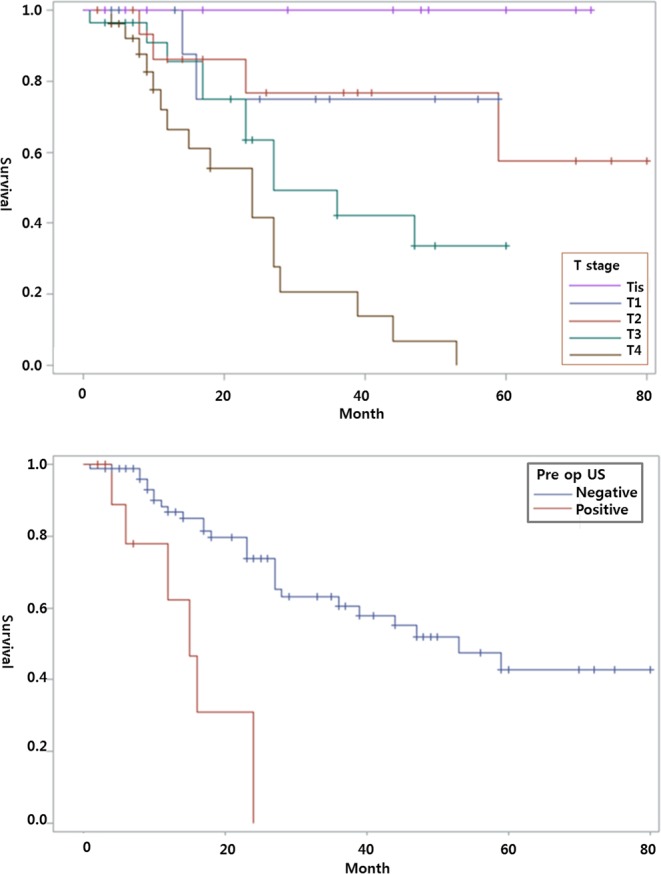
Melanoma disease-free-survival according to T stage (**a**) and ultrasound findings (**b**).

The tendency for SLN involvement increased with higher tumor stage, with rates of 8.3% (2/24) for T1, 16.7% (4/24) for T2, 29.1% (7/24) for T3, and 45.8% (11/24) for T4 (*p* < 0.05). Both univariate and multivariate analysis revealed that tumor thickness, the presence of tumor ulceration, and positive preoperative US findings of LN were significantly associated with SLN metastasis (*p* < 0.05). No significant association was found between SLN metastasis and the other pathologic features. In univariate analysis of disease-free survival, tumor thickness, LN metastasis, and positive preoperative US findings were significantly associated (*p* < 0.05). In multivariate analysis, tumor thickness (OR = 2.23, *p* = 0.0003) and LN metastasis (OR = 2.80, *p* = 0.01) were significantly associated (Fig. 1). Among 24 patients with SLN metastasis on resection, 19 patients (79.2%) showed distant metastases on follow-up (median 16 months, range 4 to 59 months). Their tumor staging was T1 (n = 2), T2 (n = 2), T3 (n = 5), and T4 (n = 10).

## Discussion

Numerous studies conducted in Western countries have revealed a predominant diagnosis of acral malignant melanomas in Asians and Hispanics^19,20^. The frequency of acral malignant melanoma based on our data (89%) was similar to the prior study (86%)^20^. Nowadays, Hispanics and Asians are present everywhere across the world. Hence, it becomes important to pay attention and to be familiar with varied features of acral malignant melanoma. The acral malignant melanoma has two different epidemiologic characteristics compared to other cutaneous melanomas in Western and Asian countries, 1) it occurs later in life, and 2) it displays no significant relationship with sunlight exposure^19–21^. Further, physical stress and the pressure imposed on the region may be important in the etiology of acral malignant melanoma^22^. Presently, the incidence of acral malignant melanoma is higher in lower limbs than in upper limbs, with the most common location being the sole, as reported in previous studies and consistent with the hypothesized importance of physical stress and trauma^20–22^. Our population also showed the similar tendency in epidemiology and the pressure imposed original region including sole, heel, and toe (75%).

The most common primary metastatic site of acral malignant melanoma in our population was the loco-regional recurrence including regional LNs and in-transit metastases (22/34, 64.7%). The other sites were the lung, distant LN, contralateral limb, scalp, and liver. The brain parenchymal metastasis was not common in acral malignant melanoma unlikely other subtypes of melanoma. It took about 21 months for developing of distant metastasis in our cohort (n = 34). Among the 34 patients, 19 patients already had SLN involvement at the time of initial diagnosis, and their distant metastasis occurred in a shorter period (median, 16 months) than others (median, 23 months). The tumor stages of 19 patients having distant metastasis were T1 (2/2, 100%), T2 (2/4, 50%), T3 (5/7, 71.4%), and T4 (10/11, 90.9%).

The accurate tumor staging of melanoma is very important. Few reports state that Breslow thickness is the most important prognostic factor for survival and occult micrometastasis can be detected by SLNB^19,20,23^. Based on our retrospective analysis of tumor itself and resected SLN, tumor thickness and the presence of tumor ulceration were observed as independent predicting factors for SLN involvement (*p* < 0.05). The correlation was better in the higher tumor stage (*p* < 0.05). However, the other clinical and pathologic factors were not influential in predicting SLN involvement. The tumor staging and LN spread on resected SLN showed significant prognostic value in disease-free survival.

Currently, there is a recommendation that SLNB should be performed in tumor stages Ib and II^19,24–27^. However, the cases having T1 tumor stage with SLN spread at the initial diagnosis in our data showed 100% distant metastasis development. Even though in the case of low tumor stage with normal preoperative US, we have to consider doing SLNB to ensure the micrometastasis of SLN. SLNB has emerged as the most powerful predictor of recurrence and survival^10^. The study by Phan *et al*.^28^, which is a comparison between the group of SLNB with immediate lymphadenectomy and the group of observation until presenting clinically evident disease, supported our data strongly. Therefore, if the pathologic results of resected SLN are positive, it would be better to treat aggressively and to take frequent follow up to prevent from delaying distant metastasis.

Preoperative US of SLNs in 98 patients had a sensitivity, specificity, PPV, and NPV of 29.2%, 94.6%, 63.6%, and 80.5%, respectively. The role of preoperative US of SLNs has been contentious^13–17,29,30^. The discrepancy between preoperative US findings of SLN and pathologic results of resected SLN was evident especially in patients with a higher tumor stage, more than 75% (*p* < 0.001). However, still, the discrepancy existed in lower tumor stage (about 25%) too. The low sensitivity of preoperative US findings of SLN suggests that the nececity of the pathologic confirmation of SLN in acral malignant melanoma patients even in the lower tumor stage^14,17^. When we consider the complications such as infection, lymphocele, and lymphedema caused by resection of SLN, US-FNA may have a role in the future diagnostic algorithm of melanoma patients^16^. For univariate and multivariate analysis, the T stage and LN metastasis are correlated with clinical significance, so it is better to do FNA than only US when the T stage is high. Further studies will be needed to conclusively determine this possibility.

This study has a number of limitations. First, as it is a retrospective study, the LN metastasis criteria were not strictly applied on the preoperative US for SLN. The preoperative US for SLN was simply categorized as normal, suspicious, and metastasis. The numbers of metastatic LNs of resected SLN at pathologic examination and preoperative US for SLN were not well matched. Finally, since this study was performed at a single institute in Korea (albeit with 10 years of data), the sample size was small compared to studies conducted in Western countries.

In conclusion, the most common primary metastatic site of acral malignant melanoma is the ipsilateral proximal limb and regional LNs. The low sensitivity of preoperative US for SLN of acral malignant melanoma highly suggests that the preoperative US is not helpful.

## Material and Methods

This study was approved by the institutional review board of ethics committee from the authors’ hospital. IRB of Samsung Medical Center number: 2016-11-090. From January 2004 through December 2014, 150 Korean patients who were diagnosed as acral melanoma involving hand, foot, finger, or toe at the single institute were retrospectively collected from a database. IRB waived the need to obtain informed consent. Exclusion criteria were 1) no pathologic information of tumor thickness due to the previous biopsy at other hospitals, 2) no information of LN metastasis confirmed by biopsy or dissection, 3) synchronous melanoma in other sites or distant metastasis at initial work-up, and 4) follow-up loss. All patients underwent US for the preoperative evaluation of SLN. All possible LNs were evaluated according to primary tumor location followed as: the ipsilateral axilla for acral malignant melanoma in upper extremities, and inguinal areas and/or popliteal areas for lower extremities.

Preoperative US findings suggestive of LN metastasis were increased peripheral perfusion, loss of central echoes, balloon-shaped lymph node^31,32^. According to these criteria, LN was classified as a normal, suspicious, or metastatic LN in the original report. The suspicious and metastatic LNs were considered as a positive finding in this study. When the preoperative US showed positive findings, patients underwent intraoperative SLNB within 2 weeks after the preoperative SLN US. For intraoperative SLNB, a lymphoscintigraphy with peritumoral injection of radionuclide was performed preoperatively. SLNs were identified using a hand-held gamma probe and by methylene blue dye injection intraoperatively. If SLNB was positive, regional lymph node dissection was followed. The pathologic data for the tumor and resected SLN, such as tumor thickness, the presence of ulceration, cell type, subtype, perineural or endolymphatic invasion were reviewed. We retrospectively reviewed all images including the US, computed tomography (CT), and positron emission tomography (PET) and clinical data. Physical examination and US were performed with 6 months interval to detect loco-regional recurrence. CT and PET were performed intermittently depending on the patient. Disease-free survival was calculated from date of intraoperative SLNB until recurrence or censored at date of last follow-up.

Univariate and multivariate analysis was performed using logistic regression model to evaluate factors associated with SLN metastasis. Univariate and multivariate analysis to determine the prognostic value of covariates regarding disease-free survival was performed using the Cox’s proportional hazard model. Sensitivity, specificity, PPV, and NPV of US were calculated with the respective 95% CIs using exact binomial confidence limits. The Cochran-Armitage trend test was used to investigate the correlation between the preoperative US for SLN and the pathologic results of resected SLN and tumor data. *P*-value < 0.05 was considered to indicate a statistical significance.
